# Alteration of neuroinflammation detected by ^18^F-GE180 PET imaging in place-conditioned rats with morphine withdrawal

**DOI:** 10.1186/s13550-021-00849-9

**Published:** 2021-10-12

**Authors:** Junpeng Li, Da Shao, Donglang Jiang, Qi Huang, Yihui Guan, Bin Lai, Jun Zhao, Fengchun Hua, Fang Xie

**Affiliations:** 1 PET Center, Huashan Hospital, Fudan University, Shanghai, 200040 China; 2 Collaborative Innovation Center for Brain Science, School of Basic Medical Sciences and Institutes of Brain Science, Fudan University, Shanghai, 200032 China; 3 Department of Nuclear Medicine, Dongfang Hospital, Tongji University, Shanghai, 200120 China; 4 Department of Nuclear Medicine, Longhua Hospital, Shanghai University of Traditional Chinese Medicine, Shanghai, 200032 China; 5 Research Center of Translation Medicine, Shanghai Children’s Hospital, Shanghai Jiao Tong University, Shanghai, China

**Keywords:** Drug addiction, Neuroinflammation, Conditioned place aversion, Translocator protein 18 kDa, Positron emission tomography

## Abstract

**Background:**

Accumulating evidence indicates that neuroinflammation (NI) significantly contributes to drug addiction, but the conversion of NI after drug withdrawal is not clear. Here, we conducted ^18^F-flutriciclamide (GE180) positron emission tomography (PET) imaging to investigate the conversion of NI during drug withdrawal and conditioning-induced aversion by measuring the change in microglial activation with ^18^F-GE180.

**Methods:**

Twelve male adult Sprague–Dawley rats were subjected to morphine withdrawal by the administration of naloxone, and six of them were used to model conditioned place aversion (CPA). ^18^F-GE180 PET imaging was performed for 11 rats on the last day of the morphine treatment phase and for 10 rats on the response assessment phase of the behavior conditioning procedure. A ^18^F-GE180 template was established for spatial normalization of each individual image, and the differential ^18^F-GE180 uptakes between the drug withdrawal (DW) group and the drug addiction (DA) group, the CPA group and the DA group, and the CPA group and the DW group were compared by a voxel-wise two-sample t test using SPM8.

**Results:**

Both the DW group and the CPA group spent less time in the conditioning cage during the post-test phase compared with the pretest phase, but only the difference in the CPA group was significant (63.2 ± 34.6 vs. − 159.53 ± 22.02, *P* < 0.005). Compared with the DA group, the uptake of ^18^F-GE180 increased mainly in the hippocampus, visual cortex, thalamus and midbrain regions and decreased mainly in the sensory-related cortices after the administration of naloxone in both the DW and CPA groups. Increased ^18^F-GE180 uptake was only observed in the mesolimbic regions after conditioned aversion compared with the DW group.

**Conclusion:**

In morphine-dependent rats, Neuroinflammation (NI) became more severe in the addiction-involved brain regions but remitted in the sensory-related brain regions after the administration of naloxone, and this NI induced by withdrawal was further aggravated after conditioned aversion formation thus may help to consolidate the withdrawal memory.

## Background

Drug addiction is mainly characterized by the repeated use of addictive drugs. Recently, accumulating evidence has indicated that glial activation, including microglia and astrocytes, and the associated neuroinflammatory signals significantly contribute to drug addiction [1, 2], while modulation of glial activation shows some drug withdrawal symptoms [3]. However, the evolution of neuroinflammation (NI) after drug withdrawal is not clear.

Place conditioning is commonly used in cue-elicited drug craving and the treatment of addiction since the rewarding/aversive effects of the drug administered with the environment play a key role in addiction treatment [4]. Steven et al. conducted a conditioned place preference (CPP) assay to detect the efficacy of dexamethasone in attenuating the augmentation of the behavioral response to cocaine and suggested that anti-inflammatory agents may be effective in normalizing the rewarding effects of cocaine [5]. Conditioned place aversion (CPA) was more commonly used in investigating the mechanism of withdrawal. Indeed, CPA induces several brain function changes such as learning-induced synaptic plasticity [6], brain-derived neurotrophic factor (BDNF) and immediate early genes (IEGs) expression [7], protein kinase A (PKA) and kappa opioid receptor (KOR) changes [8]. But the effect of CPA training on NI is less investigated.

Translocator protein 18 kDa (TSPO), formerly known as the peripheral benzodiazepine receptor, is highly expressed in phagocytic cells of the immune system, such as microglia and macrophages [9]. The radioligands of TSPO for positron emission tomography (PET) can detect NI, mainly microglial activation and proliferation, in vivo [10]. The development of ^11^C-PK11195 as the first-generation TSPO PET ligand made TSPO a surrogate marker of NI [11, 12]. However, the short half-life of ^11^C, poor signal-to-noise ratio due to high nonspecific binding limited its clinical application [12]. Due to the issues of ^11^C-PK11195 and the increased interest in NI, improved fluorine-18 labeled TSPO PET tracers, such as ^18^F-DPA-714 and ^18^F-GE180, have been developed [13, 14]. These second-generation tracers possessed improved binding affinity and better kinetics. Among them, ^18^F-GE180 has been applied in several studies for investigating the NI mechanism in multiple sclerosis [15], Alzheimer’s disease [16], stroke [14] and mild NI rats model [17]. These studies demonstrated that GE180 could be a promising TSPO tracer. Here, we chose ^18^F-GE180 (flutriciclamide) to detect the NI in conditioned behavior in vivo for better understanding of the mechanism of NI in drug addiction and aid in the search for treatments for addiction.

In this study, we constructed a digital ^18^F-GE180 template for automated whole-brain analysis of ^18^F-GE180 PET images. Then, we aimed to measure the change in NI after naloxone treatment for morphine addiction and naloxone-CPA using ^18^F-GE180 PET imaging with the new digital ^18^F-GE180 template. Considering the important role NI plays in drug addiction, and the recurrence of aversive behavior after CPA training, we hypothesized that naloxone-induced morphine-withdrawal will arise NI which accounts for aversion effects, and CPA training may aggravate the NI thus help to consolidate withdrawal memory.

## Methods

### Animals and radiopharmaceuticals

Twelve male adult Sprague–Dawley rats (220–250 g) were housed singly in a 12 h light/dark cycle with free access to water and food and controlled temperature and humidity. All experimental procedures were performed according to protocols approved by Fudan University, Shanghai, China, and followed the guidelines of the National Institutes of Health Guide for the Care and Use of Laboratory Animals as well as the ARRIVE guidelines, original version. All efforts were made to minimize animal suffering and reduce the number of animals use. ^18^F-GE180 was prepared at the PET Center, Huashan Hospital, Fudan University, under the requirement of GMP.

### Chronic morphine treatment

Male adult Sprague–Dawley rats were treated with morphine according to procedures described previously [18]. Briefly, morphine dependence was induced in rats by repeated intraperitoneal (i.p.) injections of morphine twice daily at 8:00 A.M. and 7:00 P.M. The morphine dose was progressively increased from 10 mg/kg to 40 mg/kg: day 1, 2 × 10 mg/kg; day 2, 2 × 20 mg/kg; day 3, 2 × 30 mg/kg; and days 4 and 5, 2 × 40 mg/kg. Control rats were treated with saline following the same procedure.

### Conditioned place aversion

The procedure for CPA was similar to that described previously [19, 20]. CPA took place in a three-compartment place conditioning apparatus (Med Associates, USA) with distinct visual and tactile contexts, which are readily discriminated by rats. The CPA procedure consisted of three phases: preconditioning, drug treatment and conditioning phases. In the preconditioning phase (Day 1), rats were given an initial assessment of their baseline preference to determine whether rats had a preexisting preference for any of the three compartments. They were placed in the central neutral area of the apparatus for 2 min and allowed to freely explore the apparatus for 15 min. Rats showing strong unconditioned aversion or preference for any compartment were eliminated (i.e., < 20% or ≥ 80% of the session time, the number of excluded rats did not exceed 10% of the sample). During the drug treatment phase (days 2–6), rats were treated with morphine for 5 consecutive days as described in “Chronic morphine treatment” section and assigned to the drug addiction (DA) group. In the conditioning phase (days 7–10), rats were divided into drug withdrawal (DW) group and CPA group randomly. The CPA group received a naloxone injection (0.3 mg/kg) (to precipitate morphine withdrawal) after a morphine injection (40 mg/kg) before being placed in the minor preference compartment on days 7 and 9, and on the alternate days (days 8 and 10), they received a saline injection after a morphine injection (40 mg/kg) before being placed in the opposite compartment. To distinguish conditioned responses, rats in the withdrawal group received a naloxone injection (0.3 mg/kg) after a morphine injection (40 mg/kg) in the home cage on days 7 and 9 and a saline injection after a morphine injection (40 mg/kg) in the home cage on days 8 and 10. After one day of retrieval since the conditioning phase, each rat was placed blind in the same apparatus for 15 min to assess the place aversion response on day 12 (the response assessment phase). The time that rats spent in the compartment on the preconditioning phase was recorded as the pretest time, and that on the response assessment phase was recorded as the posttest time. The CPA score was defined as the time in the minor preference compartment minus the time in the opposite compartment. Since withdrawal/aversion conditioning was performed in the minor preference compartment, the opposite compartment became a more preferred compartment at retrieval after conditioning in the conditioned withdrawal/aversion group. Therefore, the CPA score was positive in the pretest session but was negative at retrieval in the conditioned withdrawal group.

### PET scanning

Micro-PET/CT imaging was conducted as described previously using a micro-PET/CT scanner (Siemens Inc., USA) [21, 22]. Briefly, static PET/CT imaging was collected for 20 min at 40 min post intravenous injection [14] of ^18^F-GE180 (296–444 MBq/kg body weight) and at 50 min post intravenous injection of ^18^F-FDG (370–555 MBq/kg body weight). Then, the rats were positioned in a spread-supine position on the imaging bed and anesthetized by 2–3% isoflurane in medical oxygen (1–2 L/min) at room temperature with an isoflurane vaporizer (Molecular Imaging Products Company, USA) during the PET/CT procedure. PET/CT images were reconstructed using the ordered subsets expectation maximization 3D algorithm, and data were reviewed using Inveon Research Workplace software (Siemens). GE180 imaging was performed on the last day of the drug treatment phase (day 6, defined as the drug addiction group) and the response assessment phase (day 12), while FDG imaging was only performed on day 6 to assist in the establishment of the GE180 template. One animal died during the GE180 imaging on day 6, and one died during CPA training, so the final sample size of DA, DW and CPA groups was 11, 6 and 5, respectively.

### Construction of the ^18^F-GE180 PET template

**Fig. 1:**
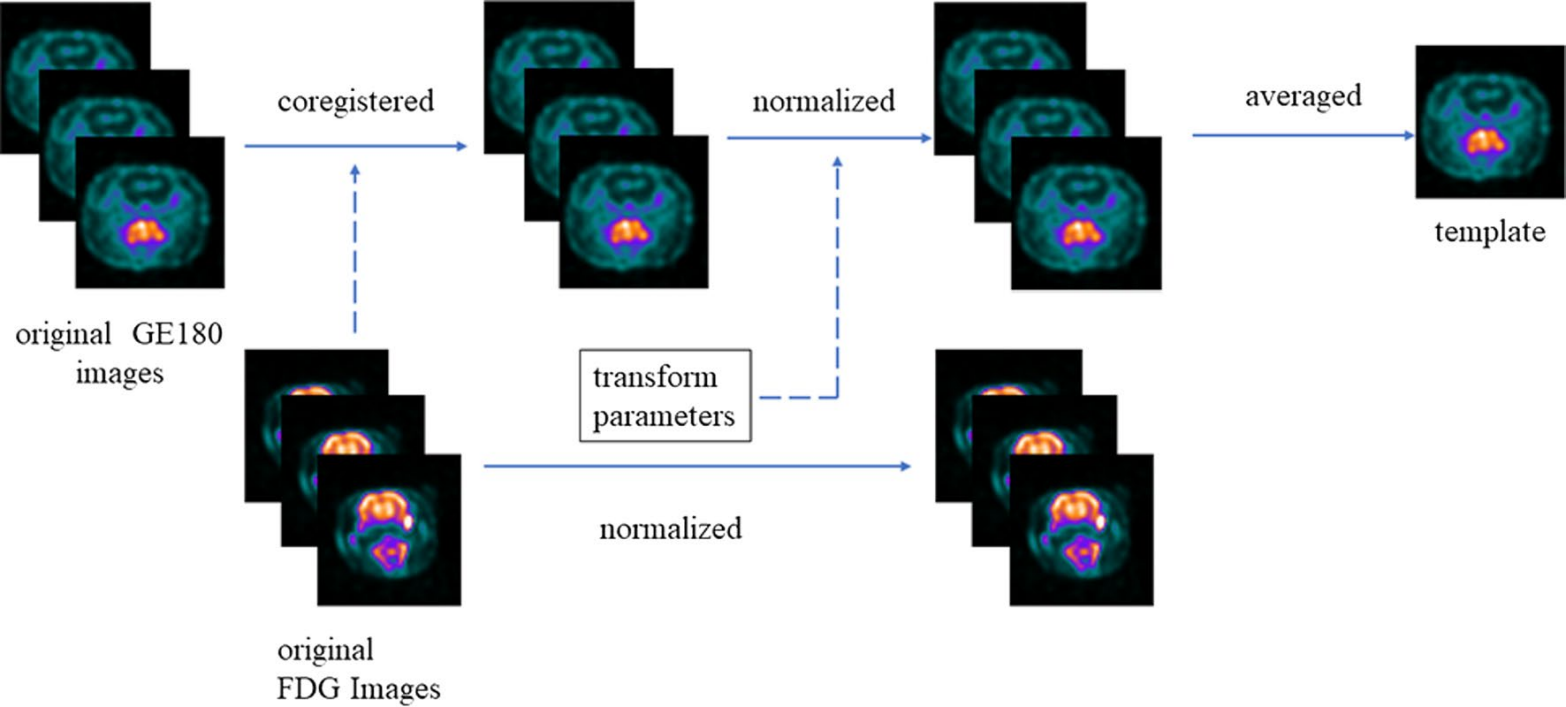
Flowchart for ^18^F-GE180 template construction. The solid arrows represent the following procedure, while the dashed arrows indicate the target for coregistration and special normalization. All of the steps were repeated only once

The rat ^18^F-GE180 PET template was reconstructed by a method described previously using SPM8 (Welcome Trust Centre for Neuroimaging, London, UK; http://www.fil.ion.ucl.ac.uk/sp) [23, 24]. Briefly, we first coregistered all of the ^18^F-GE180 images to their individual FDG images obtained on day 6 due to the low cerebral uptake of ^18^F-GE180. Then, the individual FDG images were normalized to a rat FDG template of a toolbox of SPM8 named *spmtatHEP* [25], and the transformation parameters were used for the normalization of all of the individual ^18^F-GE180 images. After that, the matrix value of the normalized ^18^F-GE180 images was rescaled to [0 255]. Finally, the normalized ^18^F-GE180 images were arithmetically averaged to create the template (Fig. 1). Since the averaged image after multiple iterations was distorted, all of the steps, including chorister, spatial normalization and arithmetic average, were repeated only once.

### Statistical analysis

Statistical analysis was conducted by GraphPad Prism 8. The normality of the distribution of the CPA scores was tested by Shapiro–Wilk normality test and Kolmogorov–Smirnov normality test. The difference of the CPA scores between multi-groups was analyzed by one-way ANOVA following by Bonferroni post hoc analysis, statistical significance was determined as *P* < 0.05.

To detect the change in the activation of glia after morphine withdrawal and CPA training, a voxel-wise two-sample t test was performed by SPM8 with global mean intensity calculation to compare the different ^18^F-GE180 uptakes between the DW group, the DA group and the CPA group in pairs using the toolbox *spmtatHEP*. A significance threshold was set as *P* < 0.001 with nonmultiple correction, and *K* > 20 voxels was set as the cluster extent threshold.

## Results

### Construction of the ^18^F-GE180 template in rats

**Fig. 2:**
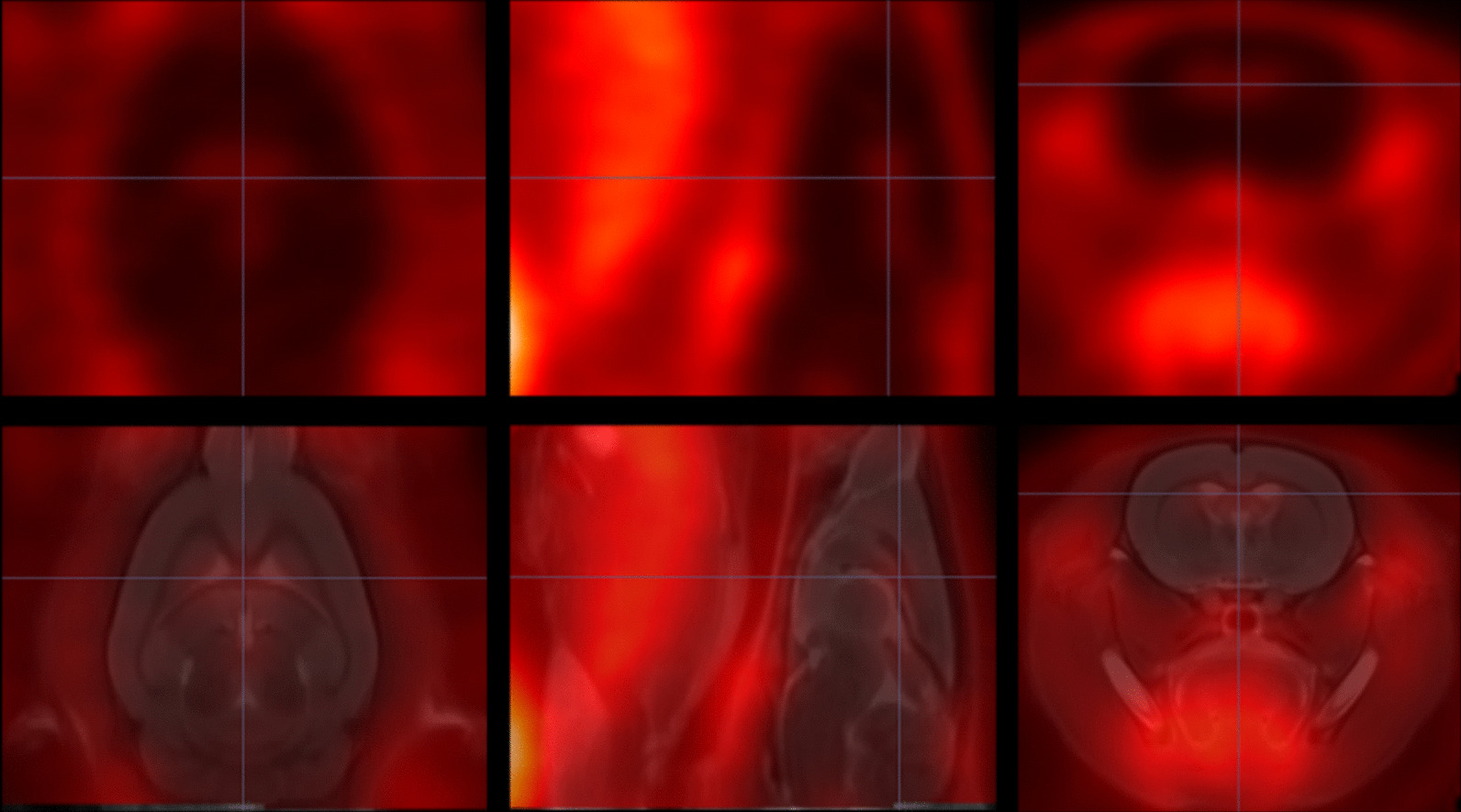
The rat GE180 template. The upper row of images is the coronal plane, sagittal plane and cross section of the template. The lower row of images is the overlaid images of the template on an MRI T2 template. The cross curve was focused on the area around the ventricle

A ^18^F-GE180 template was established (Fig. 2) for the spatial normalization of each individual image, and the coordinate of the template fitted the atlas image in Paxinos & Watson space [26]. The uptake of GE180 was mainly concentrated in the area around the ventricle and was low in the cortex area, which was consistent with previous research [27].

### Establishment of the CPA animal model

**Fig. 3:**
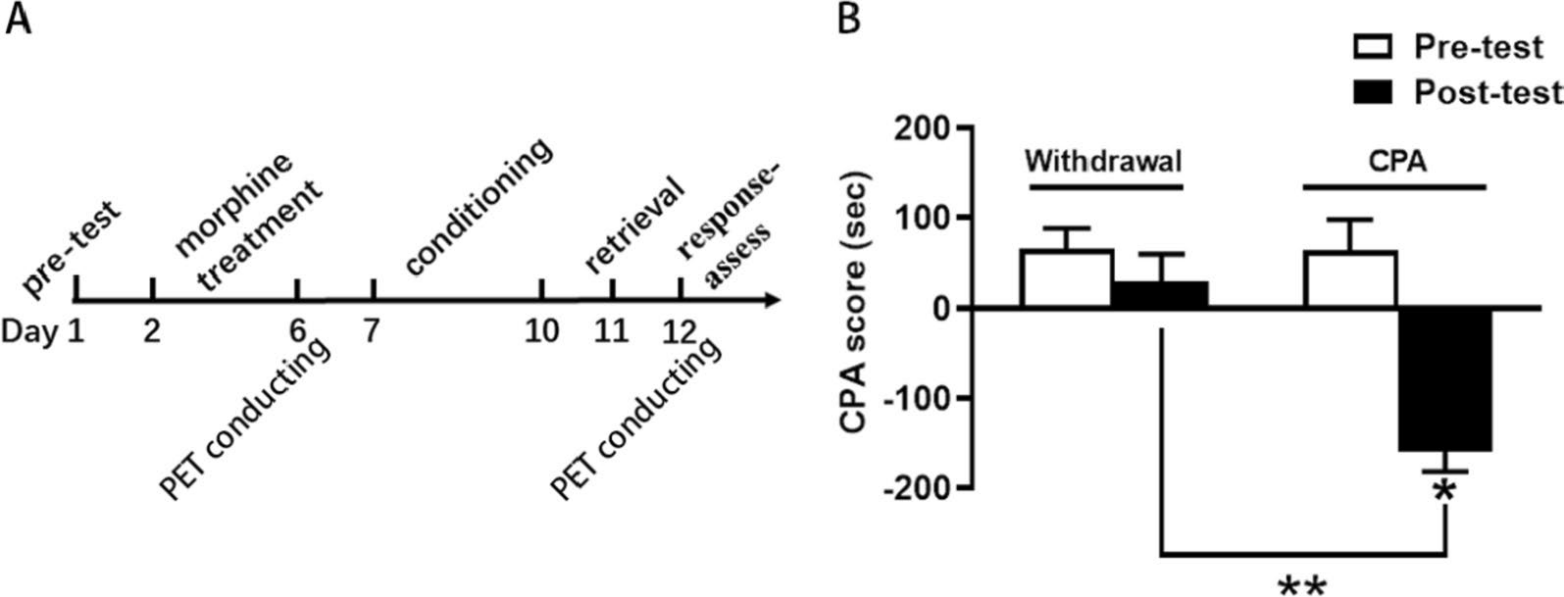
The establishment of the behavior model. **A** The timeline of the behavior produced. **B** The average CPA scores of the withdrawal group and CPA group. *P < 0.005, **P < 0.0001

As shown in Fig. 3B, after CPA training, the CPA score in the CPA group rats was significantly decreased compared with that in the withdrawal group (CPA score, − 159.53 ± 22.02 vs. 30.18 ± 28.89, *P* < 0.0001). The difference in the CPA group between the pretest and posttest was also statistically significant (CPA score, 63.2 ± 34.6 vs. − 159.53 ± 22.02, *P* < 0.005).

### Alternation of the brain uptake in different rats

#### Change in ^18^F-GE180 uptake after the administration of naloxone and conditioning training

**Fig. 4:**
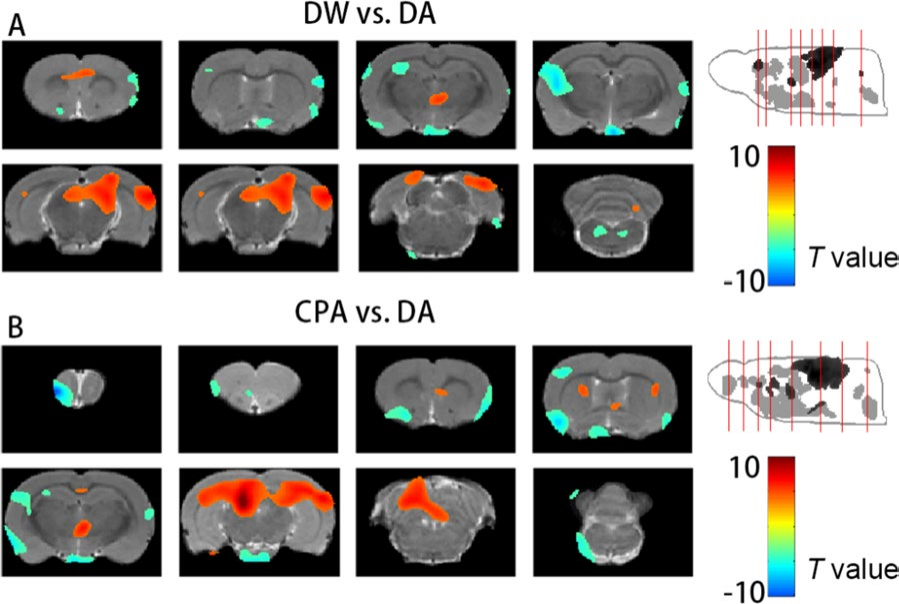
The result of the changed ^18^F-GE180 uptake after drug withdrawal and CPA formation. **A** The changed ^18^F-GE180 uptake in the DW group compared with the DA group. **B** The changed ^18^F-GE180 uptake in the CPA group compared with the DA group. The increased uptake regions are shown in red, while the decreased uptake regions are shown in blue. The red lines on the maximum intensity projection (MIP) image refer to the location of the cross section images on the left, and the cross section images are arranged from front to back, left is left. The color bar represents the *T* value of each significant voxel

The primary analysis investigated the change in the activation of glia after drug withdrawal and conditioning training (Fig. 4). After the administration of naloxone, the DW group displayed increased ^18^F-GE180 uptake in the bilateral visual and cingulate cortices, right hippocampus, thalamus and midbrain as well as decreased ^18^F-GE180 uptake in the bilateral sensory, insular and piriform cortices, optic chiasm, left hippocampus and pons compared with the DA group (Fig. 4A). Similar but wider results were found in the CPA group than in the DA group, as shown in Fig. 4B. The CPA group displayed increased ^18^F-GE180 uptake in the bilateral visual cortex, left entorhinal cortex, left septal nucleus, bilateral caudate putamen, bilateral hippocampus, thalamus, midbrain and cerebellum and decreased uptake of ^18^F-GE180 in the bilateral sensory, insular and piriform cortices, left caudate putamen, optic chiasm, olfactory and medulla compared with the DA group.

#### Increased ^18^F-GE180 uptake in the CPA group compared with the withdrawal group

**Fig. 5:**
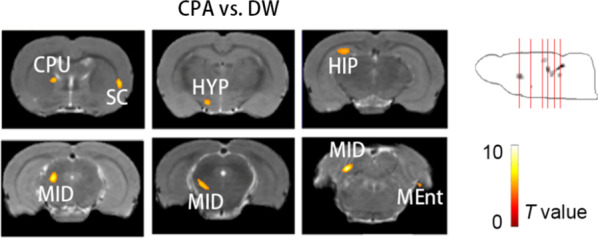
The result of the increased ^18^F-GE180 uptake in the CPA group rats compared with the withdrawal rats. The red lines on the MIP image refer to the location of the cross section images on the left, and the cross section images are arranged from front to back, left is left. The color bar represents the T value of each significant voxel. The abbreviation of each brain region is marked on the cross section images. *CPU* caudate putamen, *SC* sensory cortex, *HYP* hypothalamus, *MID* midbrain regions, *MEnt* medial entorhinal cortex

Additional analysis aimed to investigate the conversion of NI caused by withdrawal after conditioning training. Figure 5 indicates aggravated NI after conditioning training since only increased ^18^F-GE180 uptake was obtained in the right sensory cortex, right medial entorhinal cortex, left hippocampus, left caudate putamen, left hypothalamus and midbrain regions in the CPA group compared with the withdrawal group.

## Discussion

In addition to numerous studies illustrating the contribution of NI to drug addiction, we established a ^18^F-GE180 PET template and further investigated the change in microglial activation during drug withdrawal and conditioning-induced aversion in a rodent model. This is the first study to set up a ^18^F-GE180 PET template for small animals, although we did not perform any validation in the statistical analysis with this small sample size, we provided a method to set up a ^18^F-GE180 PET template for small animals.

Previous studies found that microglial activation accounts for withdrawal symptoms, and blocking the activation of microglial cells can alleviate this symptom [28, 29]. An in vitro experiment also revealed that the administration of naloxone resulted in increased expression of inflammatory mediators, including tissue necrosis factor-alpha (TNF-α), interleukin-1-beta (IL-1β), interleukin-6 (IL-6) and interleukin-10 (IL-10), in brain tissues of morphine-dependent mice [30]. Both in vivo and ex vivo studies have demonstrated that TSPO was well parallel with the microglial activation [31, 32], and PET imaging using TSPO ligands is a promising tool to visualize NI induced by the activated microglial [33–35].

In our in vivo study, despite the wider regions the CPA group exhibited than the DW group in comparison with the DA group, both groups presented overexpression of ^18^F-GE180 mainly in the hippocampus, visual cortex, thalamus and midbrain. Similar results were reported in a methadone maintenance treatment of heroin-related addiction by fMRI that the protracted abstinence patients demonstrated significantly higher brain responses in mesolimbic regions and visuospatial-attention regions [36]. The hippocampus plays an important role in memory, and morphine exposure, regardless of acute or chronic exposure, impairs hippocampus-dependent spatial learning and memory [37, 38]. The visual cortex has been demonstrated to be impacted by different behavior contexts [39] and was found to be activated during drug-cue-induced craving [40]. The thalamus, which is a vital sensory center, was found to be involved in the reinstatement, extinction and expression of drug-seeking behaviors [41, 42]. The midbrain is necessary for locomotion [43], and it has been reported that heightened midbrain activations may reduce the approach motivation for cocaine [44]. In addition, visual stimulation could induce the drug addiction by activation of mesolimbic circuits without drug ingestion, which indicates the synergistic effect of mesolimbic and visuospatial-attention on drug addiction [45]. All of these findings suggested that addiction-involved regions may be vulnerable to NI during the withdrawal stage.

More importantly, after CPA training, microglial activation was increased compared with that in the DW group. The regions mainly include the mesolimbic regions (right medial entorhinal cortex, left hippocampus, left caudate putamen, left hypothalamus and midbrain). The reduced CPA score also supported this imaging result: after conditioning training, rodents were even less willing to stay in the compartment where they received naloxone injection. Previous studies manifested that the activation of mesolimbic regions causes the release of dopamine substrates which play an important role in reward mechanism and drug reinforcement [45, 46], and drug withdrawal can cause a long-lasting decrease in dopamine release [47]. Furthermore, the medial entorhinal cortex could connect with the hippocampus for episodic memory transfer [48]. We considered that the aggravated NI that occurred in these regions after CPA training might cause the downregulation of mesolimbic dopamine system and account for the reinforcement of withdrawal memory and escape behavior. However, the connection between the mesolimbic system and other brain regions may also have a neuropsychological effect, such as the trait impulsivity and sensation-seeking that are regulated by the connection between the mesolimbic and the prefrontal cortex [49]. The connection of the nucleus accumbens with the ventral tegmental area and the lateral hypothalamus in mesolimbic system could cause hedonic feeding as well [50]. Although we did not find significant TSPO change in other regions, the NI occurred in the mesolimbic regions might involve them by other ways to influence the withdrawal behavior. Hence, in further clinical practice, mesolimbic regions are suggested for intensive monitoring in drug addiction management, and also might be a potential target for treatments to facilitate the formation of withdrawal memory.

We also detected reduced ^18^F-GE180 uptake mainly in the sensory-related cortices (the sensory, insular and piriform cortices) in both the DW and CPA group rats compared with the DA group. There are two possible reasons for this result. One is that naloxone might not only cause NI but also have an antagonistic effect on morphine [51], and previous reports have verified naloxone can reduce the activation of microglia by different manners [52, 53]. We doubted that the reduced microglia activation observed in this study might be due to the antagonistic effect on morphine. The second is that the specificity of TSPO for NI and glial cells is not exclusive. TSPO is also expressed by endothelial cells [54]; in addition, the expression of TSPO may indicate the denseness of microglial cells instead of activation [31]. Further in vitro and longitudinal studies are needed to validate these speculations.

There are also some limitations in this study. First, since low tolerance of the female rats to morphine, only male rats were included in this study. This might lead to bias of NI on drug addiction/withdrawal. The influence of sex on addiction still needs to be illustrated in further research. Second is that in this animal experiment, we did not measure the affinity of brain microglia to GE180, because there is no significant difference in the affinity of brain microglia in rats. However, when applied in clinical practice, we consider that microglia with different affinities in human brain will have an impact on the uptake of GE180, which should be taken into account.

## Conclusions

In this rodent study, we set up a ^18^F-GE180 template to investigate the conversion of NI by detecting changes in glial activation in the brains of naloxone-induced morphine withdrawal rats and CPA model rats. Specifically, after naloxone treatment, NI became more severe in the addiction-involved regions but remitted in the sensory-related regions, and this NI induced by withdrawal was further aggravated after conditioned aversion formation thus may help to consolidate the withdrawal memory. TSPO PET can well monitor the change of NI during morphine withdrawal course.

## Data Availability

All the raw data of this study, including PET images and behavior data, can be obtained through the corresponding authors on reasonable request.

## Abbreviations

CPP: Conditioned place preference
CPA: Conditioned place aversion
DA: Drug addiction
DW: Drug withdrawal
GE180: ^18^F-flutriciclamide
NI: Neuroinflammation
TSPO: Translocator protein 18 kDa
